# Assessment of Antioxidant Enzyme Superoxide Dismutase (SOD) in Oral Cancer: Systematic Review and Meta-Analysis

**DOI:** 10.1155/2024/2264251

**Published:** 2024-03-16

**Authors:** Khadijah Mohideen, Krithika Chandrasekaran, Kareema M, Jeyanthikumari T, Safal Dhungel, Snehashish Ghosh

**Affiliations:** ^1^Department of Oral Pathology and Microbiology, Sathyabama Dental College and Hospital, Sathyabama Institute of Science and Technology, Chennai 600119, India; ^2^Meenakshi Academy of Higher Education and Research, West K.K. Nagar, Chennai 600078, India; ^3^Department of Prosthodontics and Implantology, Tamil Nadu Government Dental College and Hospital, The Tamil Nadu Dr. M.G.R. Medical University, Muthusamy Salai, Chennai 600003, India; ^4^Department of Oral and Maxillofacial Surgery, College of Medical Sciences, Bharatpur 44200, Nepal; ^5^Department of Oral Pathology, College of Medical Sciences, Bharatpur 44200, Nepal

## Abstract

**Objective:**

The present article aims to comprehensively review the existing literature on superoxide dismutase (SOD) levels, an antioxidant enzyme, in oral cancer.

**Method:**

An extensive literature search was conducted across various databases, including PubMed, Wiley Online Library, Science Direct, and Cross Reference, spanning 1998–2023. At the outset, 1,177 articles were initially identified, and 907 studies were excluded due to irrelevance or duplication of the research question. Subsequently, 270 articles underwent screening evaluation, resulting in the selection of 85 articles meeting the inclusion criteria. Following this, 68 articles underwent a full-text comprehensive assessment, and ultimately, 39 were chosen for data extraction. The risk of bias in the designated articles was assessed using the Newcastle–Ottawa Scale. Finally, 13 studies were meticulously selected, offering consistent data for the ensuing meta-analysis. Meta-analysis was executed using comprehensive meta-analysis (CMA) version 3 software (Bio Stat Inc., Englewood, NJ, USA). The meta-analysis findings revealed a statistically significant decrease in SOD levels in both erythrocyte samples (*P* < 0.001) and tissue samples (*P* < 0.05) among individuals with oral cancer (OSCC) compared to the normal control group. Conversely, the analysis of three studies on salivary samples demonstrated a significant increase (*P* < 0.05) in SOD levels in the oral cancer group compared to the healthy controls.

**Conclusion:**

This systematic review underscores a statistically significant decline in SOD levels observed across diverse bio-samples in individuals with oral cancer, indicating an excess of oxidative stress (OS). Additional research is needed to delve into the relationship between SOD levels and clinic–pathological prognostic markers within the oral cancer cohort. Such investigations have the potential to significantly contribute to the development of prognostic tools grounded in OS, thereby guiding strategies for treatment planning.

## 1. Introduction

The prevailing type of head and neck cancer is oral squamous cell carcinoma (OSCC), surpassing 400,000 cases in global annual incidence [1]. The leading factors contributing to the development of OSCC are personal behaviors, such as smoking, tobacco chewing, and alcohol consumption. Additionally, a complex interplay of socioeconomic factors, environmental or occupational exposures, trauma or the presence of sharp teeth, mutations in oncogenes or tumor suppressor genes, and infections induced by oncogenic viruses could significantly contribute to the onset of oral cancer [2]. Oxidants or reactive oxygen species (ROS) are molecules with high reactivity and instability due to a single unpaired electron in their peripheral shell. Being aggressive, ROS can potentially target healthy human cells, disrupting their normal structure and function and posing a risk for malignant transformation [3]. Internally generated enzymatic and nonenzymatic antioxidants play a crucial role within the human body by neutralizing reactive species (ROS/oxidants). The protective antioxidant defense mechanism safeguards the body against the harmful effects of ROS. ROS comprise a diverse array of reactive compounds, including radical species such as superoxide anion (O_2_^−^), hydroxyl radical (OH^−^), hydroperoxyl radical (HOO^−^), and a nonradical compound known as hydrogen peroxide (H_2_O_2_) [4]. Antioxidants inhibit the formation and dissemination of free radicals [5]. When the generation of oxidants exceeds the intended levels due to excessive accumulation or reduced elimination, the resultant oxidative imbalance can lead to an insufficient supply of antioxidants. Such disproportion can trigger oxidative stress (OS), disrupting the equilibrium in the oxidant–antioxidant defense systems [6]. The compromised activity in the antioxidant defense system is a pivotal factor in the progression of various diseases. The repercussions of OS significantly contribute to irreversible damage to cellular and tissue structures, which plays a specific role in the initiation, promotion, and progression of cancer. Substantial evidence indicates that antioxidant enzymes play a crucial role in averting both the initiation and advancement of tumorigenesis [7]. Enzymatic antioxidants such as superoxide dismutase (SOD), catalase (CAT), reduced glutathione (GSSH), and glutathione peroxidase (GPx), along with nonenzymatic antioxidants like vitamins B-complex, C, E, *ß* carotene, and the mineral selenium, may be produced either by the tumor cells themselves or in response to the body's reaction to tumor growth [8].

SOD enzymes play a crucial role in managing the concentrations of diverse ROS and nitrogen species, mitigating their potential harm, and overseeing a broad spectrum of cellular processes through their signaling functions [9]. The SOD enzyme plays a vital role in regulating cell growth and is acknowledged as the primary defense mechanism against OS in aerobic cellular systems [10]. In all aerobic organisms, various SOD proteins are strategically positioned in distinct cellular and subcellular locales.

SOD counteracts two harmful substances, superoxide (O_2_^−^) and hydrogen peroxide (H_2_O_2_), converting them into water [11]. By activating the SOD enzyme, the adverse impact of the superoxide radical on the antioxidant enzyme GPx is impeded, thus preventing the subsequent deactivation of the GPx enzyme. Consequently, SOD prolongs the active phase of the GPx enzyme. The human body harbors three unique isoforms of SOD: Zn/CuSOD (SOD1) located in the cytoplasm and nucleus, MnSOD (SOD2) situated in the matrix of the mitochondrial membrane space, and Ec-SOD (SOD3) present in the extracellular space [12]. Cu/ZnSOD and MnSOD stand out as the primary antioxidant enzymes among these [13]. Despite extensive research on the involvement of the antioxidant enzyme SOD, there are uncertainties persist in the redox state of carcinogenesis [14]. The objective of the present systematic review was to assess the activities of SOD enzyme in individuals diagnosed with oral cancer and to compare with the control group of healthy individuals.

## 2. Materials and Methods

Following the prescribed PRISMA protocol [15], this systematic review has been appropriately registered in the PROSPERO database with the identifier CRD42021257722.

### 2.1. Research Hypothesis

Are there changes in the activity of the antioxidant SOD enzyme in individuals with oral squamous cell carcinoma (OSCC) compared to those in healthy groups?

Our research question adhered to the PECOS framework, emphasizing the following elements:

- Population: Patients diagnosed with oral cancer.
- Exposure: Measurement of SOD values (mean and SD) in different samples.
- Comparison: Between patients with oral cancer and healthy participants.
- Outcome: Assessing variations in SOD enzyme activities between OSCC patients and a healthy control group across various biological samples.
- Study design: Case-control and cross-sectional studies.

### 2.2. Literature Search

An extensive literature search was performed utilizing electronic databases such as PubMed, Science Direct, Wiley Online Library, and Cross Reference, including the period from 1998 to 2023. The search filtered the articles in the English language by employing MeSH terms and relevant keywords.

### 2.3. Inclusion Criteria

- Articles revealed the antioxidant status by evaluating SOD values within the OSCC group.
- The studies utilized various biosamples, presenting SOD activity values (mean and standard deviations) along with statistical significance between the OSCC group (before treatment initiation) and the control group.
- Case-control and cross-sectional studies.

### 2.4. Exclusion Criteria

- The abstracts and objectives unrelated to the research.
- The narrative, critical, systematic review articles, and case reports.
- The articles include other antioxidant enzyme markers (CAT, GPx, and GSSH) or micronutrient (antioxidant, vitamins, and minerals) assessments in the oral cancer group and they did not provide data for SOD antioxidant enzyme.
- The articles lacked adequate data (graphical representation) to compare the control and OSCC groups.
- The studies concentrated on groups with oropharyngeal or head and neck carcinomas.

### 2.5. Literature Search

Literature search of each database described in detail in Table 1.

The screening process was initiated by evaluating the titles and abstracts of the published articles. Articles meeting the inclusion criteria underwent a comprehensive full-text assessment. Three independent assessors evaluated these papers, employing the Newcastle–Ottawa Scale and scrutinizing potential limitations such as selection bias, incomplete information, data precision, and quality measures (e.g., ethical approval, informed consent, disclosure of conflicts of interest, and funding sources). The authors selected articles that met the eligibility criteria after a thorough evaluation.

### 2.6. Data Extraction

Three reviewers autonomously screened and chose the articles, and the disagreements were resolved through consensus guided by the established criteria. The selected articles underwent analysis, during which information about authorship details, publication year, cohort size, and the methodology employed to assess SOD enzyme observed values (mean and SD) for both the OSCC and control groups were extracted.

### 2.7. Meta-Analysis

The standard mean difference value was computed using comprehensive meta-analysis (CMA) version 3 software (Biostat. Englewood, NJ, USA) to create the forest plot for data analysis. The overall mean difference in SOD levels between the OSCC and control groups was determined with a 95% confidence interval. Due to substantial heterogeneity among the selected studies, a random-effect model was employed for quantitative synthesis. Articles with similar sample types, methodology, and measurement units for SOD activity levels were chosen for the quantitative analysis.

## 3. Results

**Figure 1:**
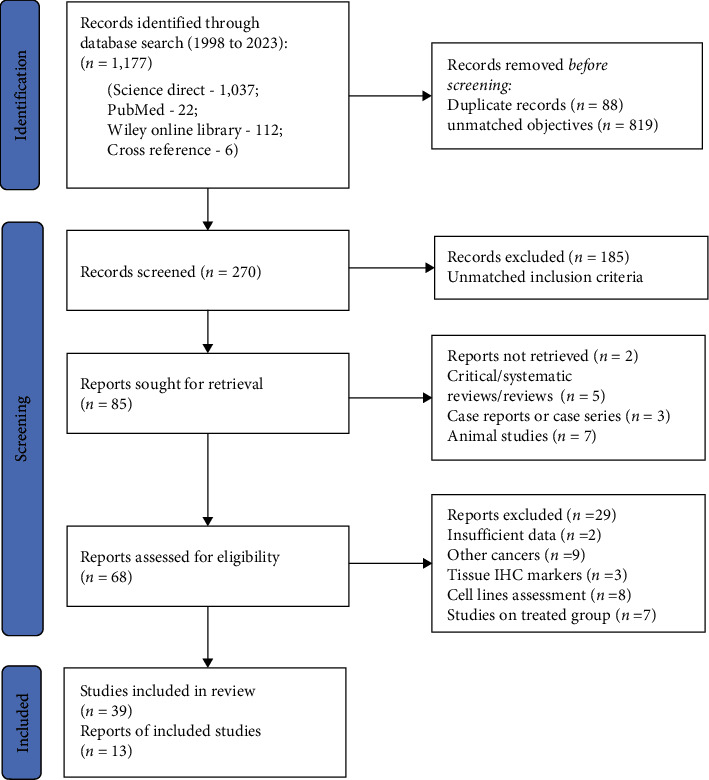
Flow chart (PRISMA) for study selection.

A total of 1,177 articles were initially identified from various search engines using the outlined search methodology. Specifically, the PubMed search yielded 22 articles, Science Direct provided 1,037 papers, Wiley Online Library contributed 112 articles, and Cross-reference offered six papers. After thoroughly analyzing search results, 907 articles were excluded for either being irrelevant to the research question or duplicative. Subsequently, 270 articles underwent screening evaluation, which led to the exclusion of 185 articles that did not meet the inclusion criteria. Out of the 85 selected articles, two were nonretrievable. The articles of critical/systematic reviews (*n* = 5), case reports or case series (*n* = 3), and animal studies (*n* = 7) were excluded from the selected articles. Following a final evaluation, 68 articles were chosen for full-text assessment. The articles with insufficient data (*n* = 2), other cancers (*n* = 9), studies on the treated group (*n* = 7), and tissue IHC and cell lines assessment (*n* = 11) were excluded during the full-text evaluation. The remaining 39 articles were identified as highly suitable for qualitative synthesis. Upon closer inspection, 13 articles with coherent data, ideal for comparison, were included in the meta-analysis (Figure 1).

**Figure 2:**
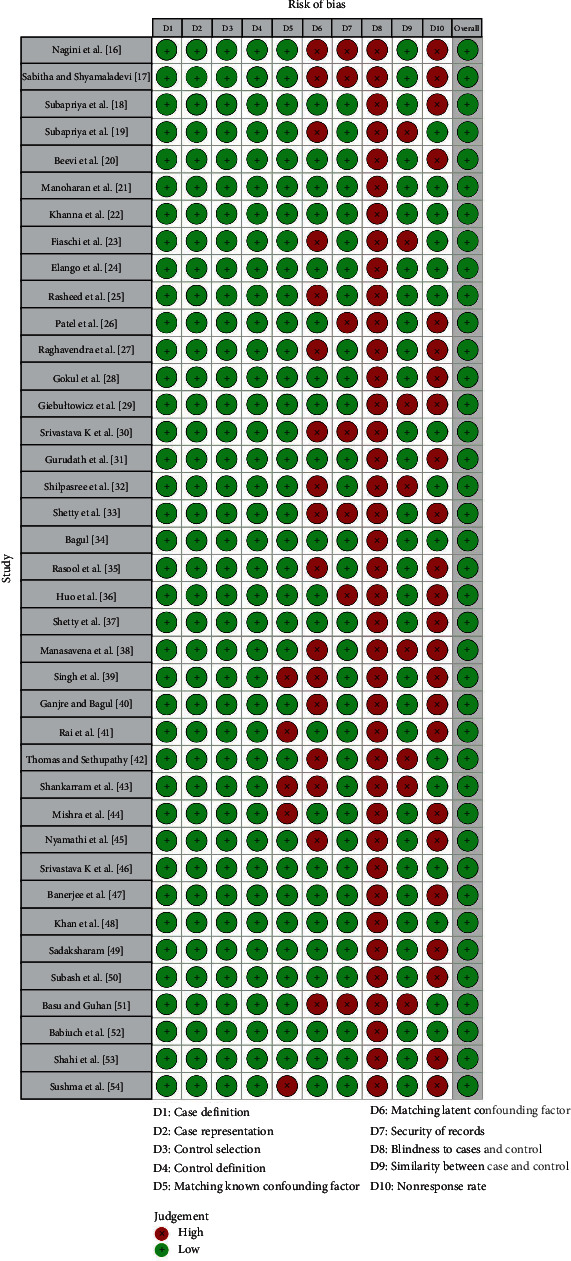
Newcastle–Ottawa quality measure for risk of bias evaluation of included studies.

**Figure 3:**
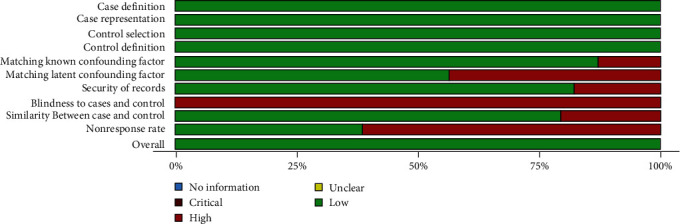
The summary of the risk of bias for the included studies.

The chosen articles were compiled, and their quality was assessed using the Newcastle–Ottawa evaluation measure as part of the qualitative analysis, as illustrated in Figure 2 [16–54]. The total score was determined by summing the awarded stars, with the interpretation as follows: 9–10 indicated excellent quality, 7–8 represented good quality, 5–6 indicated satisfactory quality, and 0–4 suggested unsatisfactory quality. Importantly, all the studies included in the analysis scored higher than 6, signifying a low risk of bias (ROB). The summary of ROB for the included studies is presented in Figure 3.

The selected studies were conducted in several nations, including IndiaClick or tap here to enter text [16–22, 24, 26–28, 30–34, 37–45, 47, 49–51, 53], Italy [23], Australia [25], Pakistan [35, 48], China [36], Poland [29, 52], and Saudi Arabia [46, 54].

The majority of studies illustrated a notable decrease in SOD levels in OSCC groups across various biological samples compared to healthy controls. In contrast, only six included studies indicated a significant increase in SOD activity levels in various samples when compared to healthy controls.

The meta-analysis findings revealed a statistically significant decrease in SOD levels in both erythrocyte samples (*P*  < 0.001) and tissue samples (*P*  < 0.05) among individuals with oral cancer (OSCC) compared to the normal control group. Conversely, the analysis of three studies on salivary samples demonstrated a significant increase (*P*  < 0.05) in SOD levels in the oral cancer group compared to the healthy controls.

The data from each included article were systematically organized and presented in Table 2 [16–54]. The authors utilized varied methods to assess the activity levels of SOD across a range of biological samples [55–65]. Gurudath et al. [31] and Nyamati et al. [45] employed the Ransel antioxidant enzyme kit for SOD level assessment in the specified biological samples and the Enzychrom™ SOD assay kit was used by Sadaksharam [49] study.

### 3.1. Meta-Analysis

**Figure 4:**
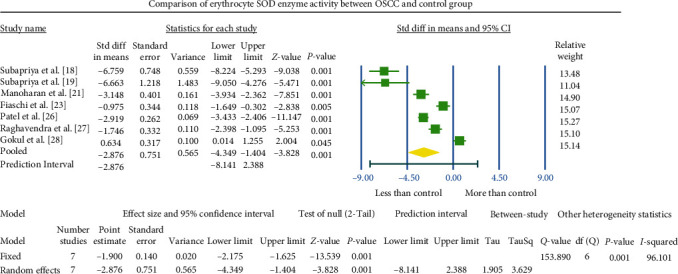
The forest plot presented the standardized mean difference (SD diff in mean) values at a 95% confidence interval, illustrating the comparison of antioxidant SOD activity in erythrocytes (ER) between the OSCC and normal control groups.

**Figure 5:**
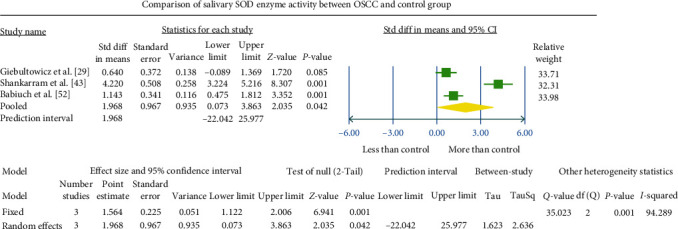
The forest plot illustrates the standardized mean difference values at 95% confidence intervals, indicating a comparison of antioxidant SOD activity levels in saliva between the OSCC and normal control groups.

**Figure 6:**
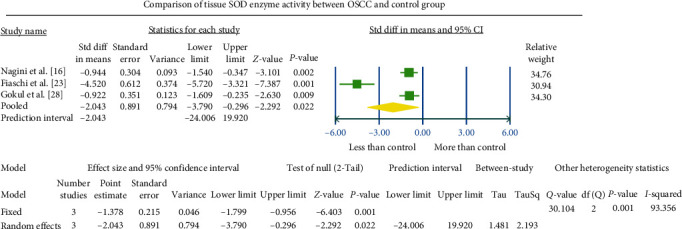
The forest plot portrays the standardized mean difference (SMD) values within 95% confidence intervals, comparing antioxidant SOD activity levels in tissue between the OSCC and normal control groups.

Various methodologies were employed to assess SOD concentration or activities in diverse biological samples. Studies providing consistent details and reporting on the same biological sample were chosen for meta-analysis. The overall observed standardized mean difference between the OSCC and control groups was −2.876 U^a^/mg Hb (95% CI −4.349 to −1.404) in the erythrocyte sample (Figure 4), 1.968 U^b^/ml (95% CI 0.073–3.863; Figure 5) in the salivary sample, and −2.043 U^a^/mg protein (95% CI −3.790 to −0.296) in the tissue sample (Figure 6).

### 3.2. Heterogeneity

The meta-analysis revealed notable heterogeneity, as indicated by the *I*^2^ values of 96.101, 94.289, and 93.356 in Figure 4–6, respectively. This substantial heterogeneity may stem from variations in the methodologies employed to assess SOD enzyme levels.

### 3.3. Publication Bias

The studies incorporated in this meta-analysis exhibited Egger's regression intercept values of −6.69, 19.77, and −11.83, with two-tailed *P* values of 0.23, 0.25, and 0.147 for erythrocyte, saliva, and tissue samples, respectively. These results indicate a low probability of publication bias in the present meta-analysis.

Merely seven studies recorded the SOD activity level in OSCC, considering clinical stages across various biological samples. In most studies, the distinctions between different stages of OSCC were deemed insignificant. Nevertheless, the reduction of SOD activity as the disease progresses from early to advanced stages proved significant in two of the included studies (*P* < 0.01; Table 3). Regarding histopathological changes, there is no discernible prediction pattern, as only three studies exhibited SOD activity changes between different histopathological grades of OSCC (Table 4) [66, 67].

## 4. Discussion

Tobacco, paan, areca nut, and other tobacco-related products directly induce irritation to the oral mucosal tissue, leading to a gradual malignant transformation. Moreover, in individuals with addictive personal habits, the delicate balance between OS and antioxidant enzymes is significantly disrupted. An intricate interplay between tobacco usage, OS-antioxidant imbalance, and genetic susceptibility may synergistically initiate carcinogenesis in individuals already exposed to predisposing factors [21–25]. Hence, the evaluation of antioxidant SOD levels can serve as a prognostic or therapeutic biomarker in OSCC [49].

The present systematic review aims to observe the antioxidant SOD enzyme activity in various biological samples for both the OSCC and healthy control groups. The review encompasses a total of 1,147 patients with oral cancer and 1,058 normal individuals assessed for SOD activity changes. The included studies employed clinical staging systems such as UICC and AJCC. The authors utilized the histopathological grading criteria proposed by Woolgar and Scott [66] and the Akhter et al. [67] method for the histopathological categorization of the OSCC patient group.

In both normal and tumorigenic conditions, SOD is recognized as a crucial antioxidant enzyme that governs cellular redox processes [68]. The impact of SOD on tumor cell growth varies based on its concentration and the host environment at the specific site [69]. The literature also indicates that patients with carcinoma exhibit significantly lower levels of antioxidant enzymes [70].

This systematic review unveiled a noteworthy decrease in the mean SOD levels across various biosamples in the OSCC group compared to the normal controls (*P*  < 0.05) [16, 17, 20–28, 30–33, 35–42, 44–48, 50, 53, 54]. Sharma et al. [3] and Bahar et al. [68] conducted studies, not included in the present systematic review due to graphical representation without actual values, that also reported a significantly lower activity of SOD in the OSCC group when compared to the normal control group. Similarly, the results of another study suggested a decrease in SOD values in erythrocyte samples of the OSCC group, although the difference did not attain statistical significance [35]. Subapriya et al. [18] observed a reduction in antioxidant levels in venous blood samples from the oral cancer group compared to the control group and at cancerous intraoral sites compared to the corresponding adjacent tissue sites. The potential explanations for reduced enzyme activity in the oral cancer group include elevated OS due to an accumulation of ROS, insufficient production of antioxidant enzymes and excessive utilization or degradation of SOD by reactive oxygen metabolites, intensive utilization of SOD to counteract superoxide anion (free radicals/ROS), and limited antioxidant capacity to neutralize ROS in a cancerous environment. Conversely, some authors reported significantly elevated SOD activity levels in the OSCC group. Specifically, one article highlighted a noteworthy increase in lymphocyte SOD levels (*P*  < 0.001) within the OSCC group [51]. Similarly, two additional studies in saliva and blood documented a significant rise in SOD levels (*P*  < 0.05) within the OSCC group compared to the control group. [29, 34, 36, 52] Another study revealed a statistically insignificant increase in SOD levels in erythrocytes of the OSCC group compared to the systemically healthy group (*P*  > 0.05) [28].

The elevated SOD activity in the OSCC samples might be attributed to the adaptive or compensatory response of cellular induction caused by an excess of O_2_^−^ (superoxide) anions resulting from higher OS and lipid peroxidation. There could be a heightened dismutation of superoxide (O_2_^−^) to H_2_O_2_ with increased SOD activity. Other antioxidant enzymes detoxify the surplus H_2_O_2_ in the blood cells [34, 51]. Studies also indicated that as the activity of other antioxidant enzymes decreases, the impact of the SOD enzyme increases [71]. Therefore, the overexpression of endogenous antioxidant enzymes is presumed to serve as a vital component of the natural antioxidative defense mechanism, which aims to scavenge lipid peroxides, contributing to the body's defense against carcinogenesis [28]. Several studies investigated SOD activity levels across different clinical stages within the OSCC group. Notably, in one study, the mean SOD levels in plasma and erythrocyte samples demonstrated a significant progressive decrease (*P*  < 0.01) as the clinical grades of OSCC advanced from stage II to stage IV [21]. Two studies reported an insignificant reduction in SOD activity levels in both plasma and tissue as the clinical stage of OSCC advanced [30, 46]. Merely three studies reported an increase in SOD enzyme activity in advanced malignant disease compared to early conditions [26, 47, 52]. One reported study observed fluctuations in the SOD level change in various stages [39]. These observations highlight the need for additional research to elucidate the role of SOD enzymes during the progression phase of the disease.

Two studies depicted a significant decrease in SOD activity levels in moderately differentiated tumors compared to well-differentiated tumors in plasma (*P*  < 0.001) [41] and erythrocyte (*P*  < 0.05) samples [26]. In contrast, a study revealed a gradual increase in salivary SOD levels as the histopathological grade of OSCC progressed. However, the extent of the difference was statistically insignificant (*P*  > 0.05) between different grades of tumors [39]. Analysis based on histopathological grades was only available in three studies. Moreover, this assessment was carried out across different samples utilizing diverse methodologies with varying units of measurement. As a result, no definitive predictions can be made concerning changes in SOD activity based on varying histopathological grades.

Fu et al. [72] reported that a higher expression of MnSOD was positively correlated with a significantly improved disease-specific survival period compared to patients with lower MnSOD expression levels (*P* − 0.009). Particularly, MnSOD overexpression was associated with favorable prognoses in individuals with moderate or poorly differentiated tumors (*P* − 0.045), clinical-stage I tumors (*P* − 0.002), and those who had undergone postoperative adjunct radiotherapy (*P* − 0.048). However, the altered levels of MnSOD expression did not predict disease-specific survival in patients with clinical stages II–IV and T2–T4 oral cancer stages. In contrast, Salzman et al. [73] and Yokoe et al. [74] proposed that OSCC patients with a markedly elevated expression of the SOD2 gene are associated with lymph node metastasis. Some studies have indicated that the SOD2-dependent expression of H_2_O_2_ results in the upregulation of MMPs (including MMP-1 and MMP-9). The association between elevated MMP levels, increased invasion, extracapsular spread (ECS), and enhanced tumor metastasis is noteworthy [75–77]. Ye et al. [78] and Liu et al. [79] highlighted that a substantial upregulation of the MMP-1 gene in tongue OSCC signifies the correlation between elevated SOD2 levels and heightened metastatic potential in OSCC. A plausible explanation for the earlier conflicting statement is that increased OS, resulting from the excessive production of H_2_O_2_, contributes to the aggressiveness of tongue squamous cell carcinoma (while concurrently elevated SOD2 expression may not be causative). In line with the previous statement, few in vivo studies have indicated that higher expression of MnSOD protects against further tumor growth in oral and cheek pouch carcinoma [80, 81]. The precise role of SODs in carcinogenesis has been extensively investigated, yet it remains unclear.

The outcomes from this meta-analysis indicate a notable reduction in SOD activity values in the OSCC group compared to the healthy control group. Specifically, in erythrocyte samples, the difference was highly significant (*P*  < 0.001), and in tissue samples, it was statistically significant (*P*  < 0.05). The overall standardized mean difference between the study and control groups was −2.876 U^a^/mg Hb (95% CI −4.349 to −1.404) in erythrocyte samples and −2.04 U^a^/mg protein (95% CI −3.79 to −0.29) in tissue samples. Conversely, three included studies of the salivary samples meta-analysis exhibited a significant increase (*P*  < 0.05) in SOD activity compared to normal controls. The overall standardized mean difference in salivary SOD value between the study and control group was 1.968 U^b^/ml (95% CI 0.073–3.863). These observations highlight that tissue, blood, and saliva components exhibit distinct biological behaviors influenced by the local environment and immune status of patients. Baseline levels of antioxidant enzymes and their responsiveness to inducibility can vary significantly based on biological samples, sample size, methodologies, host factors, disease specificity, and ethnicity. Additionally, the limited availability of studies providing coherent data for salivary SOD enzyme meta-analysis hampers the ability to assess valid changes.

The statistically significant decline in SOD levels as the disease progresses from early to advanced phases was observed in only two studies (*P*  < 0.01). Reported studies lack a specific prediction pattern concerning histopathological changes. Recognizing biological alterations in antioxidant systems may contribute to a more accurate prognosis of OSCC [82]. Predicting disease progression may be facilitated by assessing changes in SOD activity with advancing clinical stages or histological tumor grades. According to Manasaveena et al. [38] radiation therapy induces higher OS compared to chemoradiotherapy in OSCC. Thus, the detrimental effects of tumors and the adverse impact of inappropriate treatment on patients' health are highly devastating. Future studies are required to elucidate alterations in the pro-oxidant and antioxidant systems in patients not only with oral cancer but also in primary vertebral bone lesions, leptomeningeal, and other tissue metastasis across different types of solid and hematologic cancers. Doing so sheds light on the unique patterns of disease detection and progression of each kind of metastasis. Ultimately, it develops tailored treatment approaches for each cancer type [83, 84].

## 5. Conclusion

Our systematic review revealed statistically significant reductions in SOD enzyme activity across various biosamples in the oral cancer group. However, further evaluation with a larger sample size is warranted. In the current context, exploring prognostic markers such as the antioxidant enzyme SOD could enhance the selection of effective therapy, intervention methods, monitoring of therapeutic strategies, and identification of tumor resistance to improve the survival of oral cancer patients. Regular assessment of antioxidant status holds the potential to serve as a prognostic biomarker in individuals at high risk, offering benefits in reducing morbidity and mortality among OSCC patients while enhancing their quality of life.

## Figures and Tables

**Table 1:** Literature search process.

Database	Science direct	PubMed	Wiley online library
Key words free-text terms	“Superoxide dismutase” or “SOD”or “antioxidant ^*∗*^”, and “oral cancer” or “OSCC.”	“Superoxide dismutase” or “SOD” or “antioxidant ^*∗*^”, and “oral cancer” or “OSCC.” Filter: both genders Age: 19–44 and 45+ years	“Superoxide dismutase” or “SOD” or “antioxidant ^*∗*^”, and “oral cancer” or “OSCC.”

Sample type	“Saliva ^*∗*^” or “GCF”, or “serum” or “blood”	“Saliva ^*∗*^” or “GCF”, or “serum” or “blood”	“Saliva ^*∗*^” or “GCF”, or “serum” or “blood”

Access type	Research article	Human studies/abstract	All articles

Publication titles and subject areas	Medicine and dentistry	Clinical study/comparative/evaluation/observational studies	Oral diseases

Language	English	English	English

Duration	1998–2023	1998–2023	1998–2023

**Table 2:** The levels of antioxidant SOD activities or concentrations in various samples between the normal control group and patients with OSCC.

Author		OSCC group		Control group		Measurement	Method of assessment	Stat sig
Study	Sample type	Sample size	Mean ± SD	Sample size	Mean ± SD	Unit	Study	*P*-value
Nagini et al. [16]	Ti	24	13.18 ± 3.97	24	16.65 ± 3.36	U^a^/mg pr	Kakkar et al. [55]	<0.001
Sabitha et al. [17]	Er	12	4.8	12	6.3	—	Misra and Fridovich (50% reduction at auto-oxidation) [56]	<0.01
Subapriya et al. [18]	Er	24	1.35 ± 0.13	24	2.49 ± 0.2	U^a^/mg Hb	Kakkar et al. [55]	<0.05
	Ti	24	14.55 ± 1.35	24	—	U^a^/mg pr	—	—
Subapriya et al. [19]	Er	6	1.53 ± 0.22	12	3.63 ± 0.35	U^a^/mg Hb	Kakkar et al. [55]	<0.05
Beevi et al. [20]	Er	15	10.07 ± 2.93	15	21.35 ± 2.80	U^b^ /100 mg pr	Misra and Fridovich [56]	<0.001
Manoharan et al. [21]	Er	48	1.91 ± 0.1	16	2.29 ± 0.17	U^a^/mg Hb	Kakkar et al. [55]	<0.01
	Pl	48	3.27 ± 0.35	16	4.19 ± 0.31	U^a^/ml		
Khanna et al. [22]	Se	20	0.06 ± 0.12	20	0.43 ± 0.95	U/mg pr	Marklund and Marklund [57]	<0.001
Fiaschi et al. [23]	Er	18	2.86 ± 0.928	20	3.55 ± 0.422	U/mg Hb	Paoletti et al. [58]	<0.05
							Inhibition of superoxide-induced NADH oxidation	
	Ti	18	8.55 ± 1.203	20	19.37 ± 3.092	U/mg pr		<0.001
Elango et al. [24]	Er	63	3.75 ± 0.41	45	5.94 ± 0.63	U/min/mg pr	Marklund and Marklund [57]	<0.001
Rasheed et al. [25]	Er	24	7.25 ± 2.05	24	14.59 ± 1.43	U^a^/100 mg pr	Kakkar et al. [55]	<0.001
Patel et al. [26]	Er	126	2.023 ± 0.150	30	2.4709 ± 0.12	U/mg Hb	Kakkar et al. [55]	<0.05
Raghavendra et al. [27]	Er	25	5.911 ± 1.419	25	8.145 ± 1.122	U/mg Hb	Beauchamp and Fridovich [59]	<0.001
Gokul et al. [28]	Er	18	0.039 ± 0.010	25	0.0335 ± 0.01	U^c^/mg Hb	Marklund and Marklund [57]	0.053
	Ti	18	2.45 ± 1.21	18	4.15 ± 2.31	U^c^/mg pr		<0.01
Giebułtowicz et al. [29]	Sa	10	0.94 ± 0.99	30	0.6 ± 0.4	U/mg pr	Beauchamp and Fridovich [59] Based on Formation of diformazan	0.0435
	Sa	10	0.57 ± 0.07	30	0.53 ± 0.06	U/ml	—	—
Srivastava et al. [30]	Er	20	1.45 ± 0.112	20	2.280 ± 0.301	U^a^/gHb	Kakkar et al. [55]	<0.001
Gurudath et al. [31]	Cytosol &hemolysate	25	49.75 ± 7.88	25	178.4 ± 10.33	U/ml	Ransel kit (Inhibition of superoxide-induced NADH oxidation)	<0.001
Shilpasree et al. [32]	Er	30	1.57 ± 0.14	30	2.91 ± 0.35	U/mn/mg pr	Nandi et al. [60] (Inhibition of the auto-oxidation of pyrogallol)	<0.0001
Shetty et al. [33]	Sa	25	0.34	25	0.95	U/mg pr	Beauchamp and Fridovich [59]	<0.001
Bagul et al. [34]	Se	25	3.92 ± 1.75	25	3.11 ± 1.95	U^d^/ml	Marklund and Marklund [57]	0.026
Rasool et al. [35]	Er	30	0.15 ± 0.1	10	0.92 ± 1.79	ng/ml	Spectrophotometry	0.21
	Sa	30	0.61 ± 0.25	10	1.16 ± 0.1	ng/ml		<0.001
Huo et al. [36]	Er	25	0.035	25	0.028	U/mg Hb	Marklund and Marklund [57]	<0.05
	Ti	15	2	15	5	U/mg pr		<0.01
Shetty et al. [37]	Se	50	2.09 ± 0.16	65	4.34 ± 0.06	U/mg of Hb	NBT	<0.001
	Sa	50	0.07 ± 0.01	35	0.17 ± 0.03	U/mg pr		
Manasavena et al. [38]	Pl	20	34.54	20	190.4	*µ*g/dl	Sun et al. [61] (Inhibition of a superoxide-induced NADH oxidation)	—
Singh et al. [39]	Sa	50	0.027 ± 0.029	50	0.9911 ± 1.21	U/mg	Beauchamp and Fridovich [59] & Almadori et al. [62]	<0.01
Ganjre et al. [40]	Se	30	2.3243 ± 0.99	30	4.252 ± 1.949	U^d^/ml	Marklund and Marklund [57]	<0.05
Rai et al. [41]	Pl	20	58.82 ± 3.135	20	189.45 ± 14.2	—	Marklund and Marklund [57]	<0.001
Thomas et al. [42]	Pl	20	10.4 ± 2.4	20	18.28 ± 1.3	U^a^/ml	Kakkar et al. [55]	<0.05
Shankarram et al. [43]	Sa	25	4.17 ± 0.252	25	3.21 ± 0.2	U/ml	ELISA kit (Cayman)	—
Misra et al. [44]	Se	20	52.63 ± 4.02	20	189.45 ± 14.2	—	Marklund and Marklund [57]	<0.001
Nyamathi et al. [45]	Plasma hemolyse	10	47.55 ± 10.32	10	194.35 ± 14.3	U/ml	Suttle et al. [63] Ransel antioxidant enzyme kit	<0.001
Srivastava et al. [46]	Ti	20	14.28 ± 0.67	20	18.54 ± 0.54	U^a^/g Hb	Kakkar et al. [55]	<0.001
Banerjee et al. [47]	Mi	30	45.14 ± 0.88	20	98.5 ± 0.87	—	Image J—Western blot films	—
Khan et al. [48]	Se	50	0.13 ± 0.008	20	0.47 ± 0.001	ng/ml	Kakkar et al. [55]	<0.05
Sadaksharam [49]	Se	29	196.9 ± 6.215	29	226.57 ± 6.74	U/ml	Enzychrom™ assay kit	<0.001
Subash et al. [50]	Pl	35	710.2 ± 78.2	30	958.8 ± 159.9	U/g Hb	Winterbourn et al. [64]	<0.05
Basu et al. [51]	Ly	30	29.27 ± 5.31	50	15.36 ± 2.43	U/mg of pr	Misra and Fridovich [56]	<0.001
Babiuch et al. [52]	Sa	20	7.07 ± 5.3	20	2.36 ± 2.42	U^b^/ml	Misra and Fridovich [56]	0.002
Shahi et al. [53]	Er	25	4.6 ± 2.2	45	10.8 ± 7.4	U^a^ /min/10^7^ cells	Choi et al. [65]	<0.02
Sushma et al. [54]	Se	100	1.49 ± 0.49	102	4.37 ± 1.43	U^c^/100 mg pr	Marklund and Marklund [57]	<0.005

OSCC-oral squamous cell carcinoma, SD-standard deviation, Ti-tissue, Mi-mitochondria, Pl-plasma, Se-serum, Er-erythrocyte, Ly-lympholysate, Sa-saliva, Stat Sig-statistical significance, NBT-nitroblue tetrazolium, VDAC1 - voltage-dependent anion channel 1, and pr-protein. ^a^The amount of enzyme required for 50% inhibition of the formation of NADH-phenazine methosulfate NBT formazan at 520 nm. ^b^The amount of enzyme necessary to inhibit 50% epinephrine autoxidation. ^c^The amount of enzyme necessary to cause 50% inhibition of pyrogallol autoxidation. ^d^The amount of enzyme necessary to cause 50% inhibition of pyrogallol autoxidation per 30 ml of the assay mixture.

**Table 3:** The SOD enzyme level changes in different biosamples of patients in various clinical stages of OSCC.

Author	Samples		Stage I	Stage II	Stage III	Stage IV	Measure	Stat Sig	Stage
	Type	Size	Mean ± SD	Mean ± SD	Mean ± SD	Mean ± SD	Unit	*P*-value	Criteria
Manoharan et al. [21]	Pl	48	—	3.61 ± 0.72	3.2 ± 0.17	2.99 ± 0.17	U^a^/ml	<0.01	UICC
	Er	48	—	2.08 ± 0.08	1.92 ± 0.13	1.73 ± 0.09	U^a^/mg Hb	<0.01	UICC

Patel et al. [26]	Er	126	1927.15 ± 203.9		2119.5 ± 115.0		U/mg Hb	In sig	AJCC
Srivastava et al. [30]	Pl	20	—	1.52 ± 0.08	1.44 ± 0.13	1.43 ± 0.1	U^a^ /mg Hb	In sig	TNM
Singh et al. [39]	Sa	50	0.017 ± 0.014	0.037 ± 0.019	0.019 ± 0.008	0.030 ± 0.036	U/mg	0.548	TNM
Srivastava et al. [46]	Ti	20	—	14.8 ± 0.48	14.27 ± 0.4	13.97 ± 0.8	U^a^ /mg Hb	In sig	TNM
Banerjee et al. [47]	Mi	30	—	46.16 ± 0.88	16.55 ± 0.48	72.7 ± 1.29	—	—	TNM

Author	Type	Size	T1 Mean ± SD	T2 Mean ± SD	T3 Mean ± SD	T4 Mean ± SD	Unit	*P* value	Criteria

Babiuch et al. [52]	Sa	20	8.89 ± 8.68	6.08 ± 4.61	5.71 ± 3.79	11.1 ± 3.14	U^b^/ml	0.56	T Stage

OSCC-oral squamous cell carcinoma, Stat Sig-statistical significance, SD-standard deviation, Pl-plasma, Er-erythrocyte, Ti-tissue, Sa-saliva, and Mi-mitochondria. ^a^The amount of enzyme required for 50% inhibition of the formation of NADH-phenazine methosulfate NBT formazan at 520 nm. ^b^The amount of enzyme necessary to inhibit 50% epinephrine autoxidation.

**Table 4:** The SOD enzyme activity level changes in different samples of patients with various histopathological grades of OSCC.

Author	Sample		OSCC (WD)	OSCC (MD)	OSCC (PD)	Measure	Stat Sig	H/P grade
	Type	Size	Mean ± SD	Mean ± SD	Mean ± SD	Unit	*P*-value	Criteria
Patel et al. [26]	Er	126	2212.4 ± 112.3	2,137 ± 76.2	2199.5 ± 244.2	U/mg Hb	0.046	—
Singh et al. [39]	Sa	50	0.026 ± 0.035	0.027 ± 0.021	0.029 ± 0.027	U/mg	0.961	Woolgar and Scott [66]
Rai et al. [41]	Pl	20	59.22 ± 4.01	58.43 ± 2.26	—	—	<0.001	Akhter et al. [67]

OSCC-oral squamous cell carcinoma, WD-well-differentiated, MD-moderately differentiated, PD-poorly differentiated, Stat Sig-statistical significance, SD-standard deviation, Sa-saliva, Pl-plasma, and Er-erythrocyte.

## Data Availability

Data sharing does not apply to this study.

## Abbreviations

OSCC: Oral squamous cell carcinoma
SCC: Squamous cell carcinoma
ROS: Reactive oxygen species
OS: Oxidative stress
SOD: Superoxide dismutase
MMP: Matrix metallo proteinase
SR: Systematic review
UICC: Union for International Cancer Control
AJCC: American Joint Committee on Cancer.

## References

[1] Zanaruddin S. N. S., Yee P. S., Hor S. Y. (2013). Common oncogenic mutations are infrequent in oral squamous cell carcinoma of Asian origin. *PLoS One*.

[2] Mohideen K., Krithika C., Jeddy N., Bharathi R., Thayumanavan B., Sankari S. L. (2019). Meta-analysis on risk factors of squamous cell carcinoma of the tongue in young adults. *Journal of Oral and Maxillofacial Pathology*.

[3] Sharma M., Rajappa M., Kumar G., Sharma A. (2009). Oxidant-antioxidant status in Indian patients with carcinoma of posterior one-third of tongue. *Cancer Biomarkers*.

[4] Liochev S. I., Fridovich I. (2007). The effects of superoxide dismutase on H_2_O_2_ formation. *Free Radical Biology and Medicine*.

[5] Chole R. H., Patil R. N., Basak A., Palandurkar K., Bhowate R. (2010). Estimation of serum malondialdehyde in oral cancer and precancer and its association with healthy individuals, gender, alcohol, and tobacco abuse. *Journal of Cancer Research and Therapeutics*.

[6] Silva P. V. D., Troiano J. A., Nakamune A. C. M. S., Pessan J. P., Antoniali C. (2016). Increased activity of the antioxidants systems modulate the oxidative stress in saliva of toddlers with early childhood caries. *Archives of Oral Biology*.

[7] Korde S. D., Basak A., Chaudhary M., Goyal M., Vagga A. (2011). Enhanced nitrosative and oxidative stress with decreased total antioxidant capacity in patients with oral precancer and oral squamous cell carcinoma. *Oncology*.

[8] Choudhari S. K., Chaudhary M., Gadbail A. R., Sharma A., Tekade S. (2014). Oxidative and antioxidative mechanisms in oral cancer and precancer: a review. *Oral Oncology*.

[9] Panwar A., Ruhil S., Keluskar V., Jirge V. L., Kumar S. L., Sridhar M. (2023). Evaluation of superoxide dismutase, an antioxidant enzyme, in oral squamous cell carcinoma: a systematic review and meta analysis. *Journal of Datta Meghe Institute of Medical Sciences University*.

[10] Blokhina O., Virolainen E., Fagerstedt K. V. (2003). Antioxidants, oxidative damage and oxygen deprivation stress: a review. *Annals of Botany*.

[11] Sies H. (2017). Hydrogen peroxide as a central redox signaling molecule in physiological oxidative stress: oxidative eustress. *Redox Biology*.

[12] Griess B., Tom E., Domann F., Teoh-Fitzgerald M. (2017). Extracellular superoxide dismutase and its role in cancer. *Free Radical Biology and Medicine*.

[13] Fukai T., Ushio-Fukai M. (2011). Superoxide dismutases: role in redox signaling, vascular function, and diseases. *Antioxidants & Redox Signaling*.

[14] Liu X., Wang A., Muzio L. L. (2010). Deregulation of manganese superoxide dismutase (SOD2) expression and lymph node metastasis in tongue squamous cell carcinoma. *BMC Cancer*.

[15] Page M. J., McKenzie J. E., Bossuyt P. M. (2021). The PRISMA 2020 statement: an updated guideline for reporting systematic reviews. *Systematic Reviews*.

[16] Nagini S., Manoharan S., Ramachandran C. R. (1998). Lipid peroxidation and antioxidants in oral squamous cell carcinoma. *Clinica Chimica Acta*.

[17] Sabitha K. E., Shyamaladevi C. S. (1999). Oxidant and antioxidant activity changes in patients with oral cancer and treated with radiotherapy. *Oral Oncology*.

[18] Subapriya R., Kumaraguruparan R., Ramachandran C. R., Nagini S. (2002). Oxidant-antioxidant status in patients with oral squamous cell carcinomas at different intraoral sites. *Clinical Biochemistry*.

[19] Subapriya R., Kumaraguruparan R., Nagini S., Thangavelu A. (2008). Oxidant-antioxidant status in oral precancer and oral cancer patients. *Toxicology Mechanisms and Methods*.

[20] Beevi S. S. S., Rasheed A. M. H., Geetha A. (2004). Evaluation of oxidative stress and nitric oxide levels in patients with oral cavity cancer. *Japanese Journal of Clinical Oncology*.

[21] Manoharan S., Kolanjiappan K., Suresh K., Panjamurthy K. (2005). Lipid peroxidation & antioxidants status in patients with oral squamous cell carcinoma. *The Indian journal of medical research*.

[22] Khanna R., Thapa P. B., Khanna H. D., Khanna S., Khanna A. K., Shukla H. S. (2005). Lipid peroxidation and antioxidant enzyme status in oral carcinoma patients. *Kathmandu University Medical Journal (KUMJ)*.

[23] Fiaschi A. I., Cozzolino A., Ruggiero G., Giorgi G. (2005). Glutathione, ascorbic acid and antioxidant enzymes in the tumor tissue and blood of patients with oral squamous cell carcinoma. *European review for medical and pharmacological sciences*.

[24] Elango N., Samuel S., Chinnakkannu P. (2006). Enzymatic and non-enzymatic antioxidant status in stage (III) human oral squamous cell carcinoma and treated with radical radio therapy: influence of selenium supplementation. *Clinica Chimica Acta*.

[25] Rasheed M. H., Beevi S. S., Rajaraman R., Bose S. J. C. (2007). Alleviation of oxidative and nitrosative stress following curative resection in patient with oral cavity cancer. *Journal of Surgical Oncology*.

[26] Patel J. B., Shah F. D., Shukla S. N., Shah P. M., Patel P. S. (2009). Role of nitric oxide and antioxidant enzymes in the pathogenesis of oral cancer. *Journal of Cancer Research and Therapeutics*.

[27] Raghavendra U., D’Souza V., D’Souza B. (2010). Erythrocyte malondialdeyde and antioxidant status in oral squamous cell carcinoma patients and tobacco chewers/smokers. *Biomedical Research*.

[28] Gokul S., Patil V. S., Jailkhani R., Hallikeri K., Kattappagari K. K. (2010). Oxidant-antioxidant status in blood and tumor tissue of oral squamous cell carcinoma patients. *Oral Diseases*.

[29] Giebułtowicz J., Wroczyński P., Samolczyk-Wanyura D. (2011). Comparison of antioxidant enzymes activity and the concentration of uric acid in the saliva of patients with oral cavity cancer, odontogenic cysts and healthy subjects. *Journal of Oral Pathology & Medicine*.

[30] Srivastava K. C., Austin R. D., Shrivastava D., Sethupathy S., Rajesh S. (2012). A case control study to evaluate oxidative stress in plasma samples of oral malignancy. *Contemporary Clinical Dentistry*.

[31] Gurudath S., Ganapathy K. S., D. S., Pai A., Ballal S., Asha M. L. (2012). Estimation of superoxide dismutase and glutathione peroxidase in oral submucous fibrosis, oral leukoplakia and oral cancer—a comparative study. *Asian Pacific Journal of Cancer Prevention*.

[32] Shilpasree A. S., Kumar K., Itagappa M., Ramesh G. (2013). Study of oxidative stress and antioxidant status in oral cancer patients. *International Journal of Oral & Maxillofacial*.

[33] Shetty S., Gogineni S., Kumari S., Karikal A., Shetty P., Hegde S. (2013). Salivary superoxide dismutase levels in oral leukoplakia and oral squamous cell carcinoma; a clinicopathological study. *Oxidants and Antioxidants in Medical Science*.

[34] Bagul D. N. (2013). Serum levels of antioxidant in patients with oral squamous cell carcinoma: a preliminary study. *IOSR Journal of Dental and Medical Sciences*.

[35] Rasool M., Khan S. R., Malik A. (2014). Comparative studies of salivary and blood sialic acid, lipid peroxidation and antioxidative status in oral squamous cell carcinoma (OSCC). *Pakistan Journal of Medical Sciences*.

[36] Huo W., Li Z. M., Pan X. Y., Bao Y. M., An L. J. (2014). Antioxidant enzyme levels in pathogenesis of oral squamous cell carcinoma (OSCC). *Drug Research*.

[37] Shetty S. R., Babu S., Kumari S., Shetty P., Hegde S., Castelino R. (2014). Status of salivary lipid peroxidation in oral cancer and precancer. *Indian Journal of Medical and Paediatric Oncology*.

[38] Manasaveena V., Akula K. K., Sangram V. (2014). A comparative evaluation of enzymatic antioxidant levels in pre and post therapy patients with oral cancer. *International Journal of Pharmacy and Pharmaceutical Sciences*.

[39] Singh H., Shetty P., Shreelatha S. V., Patidar M. (2014). Analysis of salivary antioxidant levels in different clinical staging and histological grading of oral squamous cell carcinoma: noninvasive technique in dentistry. *Journal of Clinical and Diagnostic Research*.

[40] Ganjre A. P., Bagul N. (2014). Estimation and comparison of oxidative stress marker superoxide dismutase and glutathione peroxidase in oral leukoplakia and oral squamous cell carcinoma. *Indian Journal of Stomatology*.

[41] Rai S., Sharma A., Ranjan V., Misra D., Panjwani S. (2015). Estimation of serum antioxidant enzymes in histopathological grades of oral leukoplakia, oral submucous fibrosis, and oral cancer: a clinicopathologic study. *Journal of Indian Academy of Oral Medicine and Radiology*.

[42] Thomas S. A., Sethupathy S. (2015). Evaluation of oxidative stress in patients with oral squamous cell carcinoma. *International Journal of Pharma and Bio Sciences*.

[43] Shankarram V., Lakshminarayanan L., Selvan T., Sudhakar U., Joysonmoses J., Parthiban S. (2015). Detection of oxidative stress in periodontal disease and oral cancer. *Biomedical and Pharmacology Journal*.

[44] Misra D., Rai S., Panjwani S., Sharma A., Singh N. (2016). Role of antioxidants as a stress factor for potentially malignant, malignant disorders and healthy individuals: a correlative study. *Journal of Dr. NTR University of Health Sciences*.

[45] Nyamati S. B., HB A., Tripathi J., Sinha N., Roy S., Agrawal R. (2016). Evaluation of serum antioxidant enzymes in oral submucous fibrosis and oral squamous cell carcinoma: a clinical and biochemical study. *Journal of Advanced Medical and Dental Sciences Research*.

[46] Srivastava K. C., Austin R. D., Shrivastava D. (2016). Evaluation of oxidant-antioxidant status in tissue samples in oral cancer: a case control study. *Dental Research Journal*.

[47] Banerjee S., Mukherjee S., Mitra S., Singhal P. (2017). Altered expression of mitochondrial antioxidants in oral squamous cell carcinoma. *Journal of Oral Science*.

[48] Khan S. R., Malik A., Ashraf M. A. B. (2017). Implication of prophetic variables and their role in the development of oral squamous cell carcinoma (OSCC). *Biomedical Research*.

[49] Sadaksharam J. (2018). Significance of serum nitric oxide and superoxide dismutase in oral submucous fibrosis and squamous cell carcinoma: a comparative study. *Contemporary Clinical Dentistry*.

[50] Subash P., Jayanthi R. (2018). Comet assay and urinary 8-OHdG: a marker of oxidative stress in oral cancer with Puducherry population. *Journal of Medical Science And clinical Research*.

[51] Basu S., Guhan N. V. (2018). Medplus. *Research & Publication*.

[52] Babiuch K., Bednarczyk A., Gawlik K. (2019). Evaluation of enzymatic and non-enzymatic antioxidant status and biomarkers of oxidative stress in saliva of patients with oral squamous cell carcinoma and oral leukoplakia: a pilot study. *Acta Odontologica Scandinavica*.

[53] Shahi Y., Samadi F. M., Mukherjee S. (2020). Plasma lipid peroxidation and antioxidant status in patients with oral precancerous lesions and oral cancer. *Oral Science International*.

[54] Sushma P. S., Jamil K., Udaykumar P. (2021). Analysis of CCND1 protein and circulatory antioxidant enzyme activity association in oral squamous cell carcinoma. *Saudi Journal of Biological Sciences*.

[55] Kakkar P., Das B., Viswanathan P. N. (1984). A modified spectrophotometric assay of superoxide dismutase. *Indian Journal of Biochemistry & Biophysics*.

[56] Misra H. P., Fridovich I. (1972). The role of superoxide anion in the autoxidation of epinephrine and a simple assay for superoxide dismutase. *Journal of Biological Chemistry*.

[57] Marklund S., Marklund G. (1974). Involvement of the superoxide anion radical in the autoxidation of pyrogallol and a convenient assay for superoxide dismutase. *European Journal of Biochemistry*.

[58] Paoletti F., Aldinucci D., Mocali A., Caparrini A. (1986). A sensitive spectrophotometric method for the determination of superoxide dismutase activity in tissue extracts. *Analytical Biochemistry*.

[59] Beauchamp C., Fridovich I. (1971). Superoxide dismutase: Improved assays and an assay applicable to acrylamide gels. *Analytical Biochemistry*.

[60] Nandi A., Chatterjee I. B. (1988). Assay of superoxide dismutase activity in animal tissues. *Journal of Biosciences*.

[61] Sun Y., Oberley L. W., Li Y. (1988). A simple method for clinical assay of superoxide dismutase. *Clinical Chemistry*.

[62] Almadori G., Bussu F., Galli J. (2007). Salivary glutathione and uric acid levels in patients with head and neck squamous cell carcinoma. *Head & Neck*.

[63] Suttle N. F., McMurray C. H. (1983). Use of erythrocyte copper: zinc superoxide dismutase activity and hair or fleece copper concentrations in the diagnosis of hypocuprosis in ruminants. *Research in Veterinary Science*.

[64] Winterbourn C. C., Hawkins R. E., Brian M., Carrell R. W. (1975). The estimation of red cell superoxide dismutase activity. *The Journal of laboratory and Clinical Medicine*.

[65] Choi H. S., Kim J. W., Cha Y. N., Kim C. (2006). A quantitative nitroblue tetrazolium assay for determining intracellular superoxide anion production in phagocytic cells. *Journal of Immunoassay and Immunochemistry*.

[66] Woolgar J. A., Scott J. (1995). Prediction of cervical lymph node metastasis in squamous cell carcinoma of the tongue/floor of mouth. *Head & Neck*.

[67] Akhter M., Hossain S., Rahman Q. B., Molla M. R. (2011). A study on histological grading of oral squamous cell carcinoma and its co-relationship with regional metastasis. *Journal of Oral and Maxillofacial Pathology*.

[68] Bahar G., Feinmesser R., Shpitzer T., Popovtzer A., Nagler R. M. (2007). Salivary analysis in oral cancer patients. *Cancer*.

[69] Inoue T., Suzuki-Karasaki Y. (2013). Mitochondrial superoxide mediates mitochondrial and endoplasmic reticulum dysfunctions in TRAIL-induced apoptosis in Jurkat cells. *Free Radical Biology and Medicine*.

[70] Shetty K. S. R., Kali A., Rachan Shetty K. S. (2015). Serum total antioxidant capacity in oral carcinoma patients. *Pharmacognosy Research*.

[71] Topdag S., Aslaner A., Tataroglu C., Ilce Z. (2005). Evaluation of antioxidant capacity in lung carcinoma. *Indian Journal of Thoracic and Cardiovascular Surgery*.

[72] Fu T. Y., Hou Y. Y., Chu S. T. (2011). Manganese superoxide dismutase and glutathione peroxidase as prognostic markers in patients with buccal mucosal squamous cell carcinomas. *Head & Neck*.

[73] Salzman R., Pácal Lás, Tomandl J. (2009). Elevated malondialdehyde correlates with the extent of primary tumor and predicts poor prognosis of oropharyngeal cancer. *Anticancer Research*.

[74] Yokoe H., Nomura H., Yamano Y. (2009). Characterization of intracellular superoxide dismutase alterations in premalignant and malignant lesions of the oral cavity: correlation with lymph node metastasis. *Journal of Cancer Research and Clinical Oncology*.

[75] Yang J., Lam E. W. N., Hammad H. M., Oberley T. D., Oberley L. W. (2002). Antioxidant enzyme levels in oral squamous cell carcinoma and normal human oral epithelium. *Journal of Oral Pathology & Medicine*.

[76] Nelson K. K., Melendez J. A. (2004). Mitochondrial redox control of matrix metalloproteinases. *Free Radical Biology and Medicine*.

[77] Zhou Y., Huang S., Shen H., Ma M., Zhu B., Zhang D. (2017). Detection of glutathione in oral squamous cell carcinoma cells with a fluorescent probe during the course of oxidative stress and apoptosis. *Journal of Oral and Maxillofacial Surgery*.

[78] Ye H., Wang A., Lee B.-S. (2008). Proteomic based identification of manganese superoxide dismutase 2 (SOD2) as a metastasis marker for oral squamous cell carcinoma. *Cancer Genomics & Proteomics*.

[79] Liu Z., Li S., Cai Y. (2012). Manganese superoxide dismutase induces migration and invasion of tongue squamous cell carcinoma via H_2_O_2_-dependent Snail signaling. *Free Radical Biology and Medicine*.

[80] Liu R., Oberley T. D., Oberley L. W. (1997). Transfection and expression of MnSOD cDNA decreases tumor malignancy of human oral squamous carcinoma scc-25 cells. *Human Gene Therapy*.

[81] Lam E. W. N., Hammad H. M., Zwacka R. (2000). Immunolocalization and adenoviral vector-mediated manganese superoxide dismutase gene transfer to experimental oral tumors. *Journal of Dental Research*.

[82] Gupta M., Gupta M., Aggarwal A., Ahuja R. (2013). Recent advancements in the diagnosis of oral premalignant and malignant lesions: a comprehensive review. *Clinical Cancer Investigation Journal*.

[83] Nguyen A., Nguyen A., Dada O. T. (2023). Leptomeningeal metastasis: a review of the pathophysiology, diagnostic methodology, and therapeutic landscape. *Current Oncology*.

[84] Chalamgari A., Valle D., Palau Villarreal X. (2023). Vertebral primary bone lesions: review of management options. *Current Oncology*.

